# Cloning, Expression and 3D Structure Prediction of Chitinase from *Chitinolyticbacter* *meiyuanensis* SYBC-H1

**DOI:** 10.3390/ijms17060825

**Published:** 2016-05-26

**Authors:** Zhikui Hao, Hangui Wu, Meiling Yang, Jianjun Chen, Limin Xi, Weijie Zhao, Jialin Yu, Jiayang Liu, Xiangru Liao, Qingguo Huang

**Affiliations:** 1 Institute of Applied Biotechnology, Taizhou Vocational & Technical College, Taizhou 318000, China; haozhikui123@126.com (Z.H.); evoh123@126.com (M.Y.); cjjkey@126.com (J.C.); xilm@tzvtc.com (L.X.); zhaowj79@126.com (W.Z.); 2 Department of Crop and Soil Sciences, University of Georgia, Griffin, GA 30223, USA; yu3577@uga.edu; 3 Bioengineering Division, Huanghuai University, Zhumadian 463000, China; jyliu@uga.edu; 4 The Key Laboratory of Industrial Biotechnology, Ministry of Education, School of Biotechnology, Jiangnan University, Wuxi 214122, China

**Keywords:** chitinase, purification, recombinant, 3D structure prediction, *Chitinolyticbactermeiyuanensis*, SYBC-H1

## Abstract

Two *CHI* genes from *Chitinolyticbacter* *meiyuanensis* SYBC-H1 encoding chitinases were identified and their protein 3D structures were predicted. According to the amino acid sequence alignment, *CHI1* gene encoding 166 aa had a structural domain similar to the GH18 type II chitinase, and *CHI2* gene encoding 383 aa had the same catalytic domain as the glycoside hydrolase family 19 chitinase. In this study, *CHI2* chitinase were expressed in *Escherichia coli* BL21 cells, and this protein was purified by ammonium sulfate precipitation, DEAE-cellulose, and Sephadex G-100 chromatography. Optimal activity of *CHI2* chitinase occurred at a temperature of 40 °C and a pH of 6.5. The presence of metal ions Fe^3+^, Fe^2+^, and Zn^2+^ inhibited CHI2 chitinase activity, while Na^+^ and K^+^ promoted its activity. Furthermore, the presence of EGTA, EDTA, and β-mercaptoethanol significantly increased the stability of CHI2 chitinase. The CHI2 chitinase was active with *p*-NP-GlcNAc, with the *K*_m_ and *V*_m_ values of 23.0 µmol/L and 9.1 mM/min at a temperature of 37 °C, respectively. Additionally, the CHI2 chitinase was characterized as an *N*-acetyl glucosaminidase based on the hydrolysate from chitin. Overall, our results demonstrated CHI2 chitinase with remarkable biochemical properties is suitable for bioconversion of chitin waste.

## 1. Introduction

Chitin is one of the most underutilized yet potentially important biomass resources on earth [1]. Chitin is stable and insoluble in water, dilute alkali, dilute acids, or most other solvents. However, all kinds of its derivatives such as *N*-acetylglucosamine, toluenesulfonyl, and chitin iodide are soluble. *N*-acetylglucosamine and chitosan can be modified chemically by their amino and hydroxyl groups, resulting in many derivatives. Such derivatives have attracted wide attention because of their potential applications in food, medicine, cosmetics, health products, biocontrol, environmental protection, scientific research, and other industries [2,3,4]. Chitin is mainly obtained from aquatic waste. Conventionally, chitin is hydrolyzed to oligo or monomeric compounds (*i.e.*, chitosan or *N*-acetylglucosamine) under acidic or alkali conditions, which usually have low yield, cause environmental pollution, and have high processing costs. Biochemical de-polymerization is a promising alternative to convert chitin.

Chitinases (E.C.3.2.1.14), the enzymes capable of hydrolyzing chitin to its oligo and monomeric components [5], are found in a wide range of organisms including viruses, bacteria, fungi, yeast, protozoans, coelenterates, nematodes, molluscs, arthropods, insects, higher plants, animals, and also in human beings [6]. There are two major categories in chitinase, endochitinases (EC 3.2.1.14) and exochitinases. Endochitinases cleave chitin randomly atinternal sites, generating low molecular mass multimers of *N*-acetylglucosamine (GlcNAc), such as chitotetraose, chitotriose, and diacetyl chitobiose. There are two subcategories of exochitinases: chitobiosidases (EC 3.2.1.29) and β-(1,4) *N*-acetyl glucosaminidases (EC 3.2.1.30). Chitobiosidases catalyze the progressive release of diacetyl chitobiose starting at the nonreducing end of chitin microfibril. Moverover, glucosaminidases cleave the oligomeric products of endochitinases and chitobiosidases, generating GlcNAc monomers. Another enzyme chitosanase (EC 3.2.1.132) is also involved in deacetylating chitin to chitosan that can be further converted to glucosamine residues by the action of chitosanase [6].

According to the similarity of amino acid sequence, six classes of chitinases have been proposed, which can be grouped into three families of glycoside hydrolases, *i.e.*, family 18, 19 and 20 [6]. Most chitinases belong to family 18, which are found in bacteria, fungi, viruses, animals, and some plants [7]. Chitinases of the three families have completely different 3-dimensional structures and molecular mechanisms, and they are likely to have evolved from different ancestors [8]. Recently, a number of attempts have been made to clone chintinase-encoding genes from several microorganisms such as *Bacillus circulans* [9], *Streptomyces thermophiles* [10], and *Serratiamar cescens* [11] to heterologously express into *E. coli*.

In our previous study, a novel chitin-degrading bacterium, *Chitinolyticbacter* *meiyuanensis* SYBC-H1, has been isolated from soil [12] that exhibited great potential in chitinase productivity for industrial application [13,14]. There is little information, however, about the structural characteristics of this chitinase. In this study, we report the cloning of the *CHI* gene from *C. meiyuanensis* SYBC-H1, 3D structural prediction of *CHI* chitinase, and its overexpression in *E. coli* BL21 cells.

## 2. Results and Discussion

### 2.1. Sequence Alignment with Chitinase

The designed primers were based on the chitinase gene sequence from bacteria of close genetic distance obtained from NCBI and two variants of *CHI* gene were cloned. *CHI1* was 498 bp which was predicted to encode aa166 amino acid polypeptide with a calculated molecular weight of approximately 17.7 kDa. *CHI2* was 1149 bp which was predicted to encode a 383 amino acid polypeptide with a calculated molecular weight of approximately 41.9 kDa.

**Figure 1:**
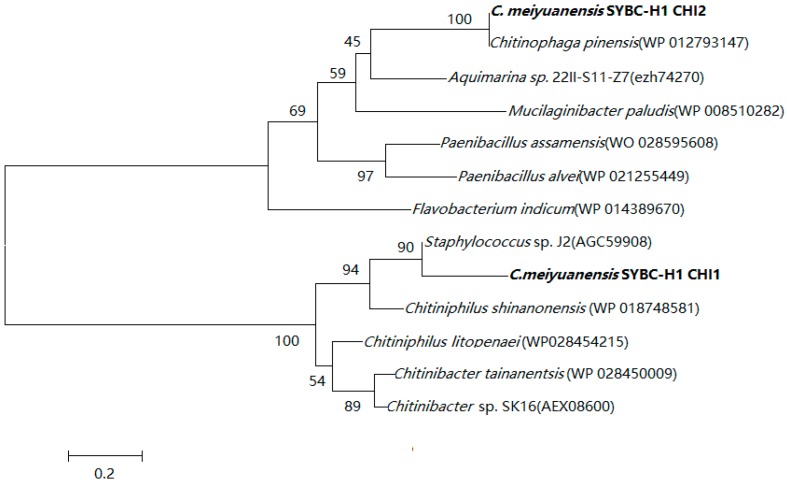
Phylogenetic tree for CHI protein of *Chitinolyticbacter* *meiyuanensis* SYBC-H1.

In order to investigate the evolutionary relationship of *C. meiyuanensis* SYBC-H1 CHI1 and CHI2 protein, a phylogenetic tree was constructed with the protein sequences of other known chitinase from the blast program (Figure 1). Phylogenetic analysis demonstrated that *C. meiyuanensis* SYBC-H1 CHI1 protein had 90% identity to chitinase from *Staphylococcus* sp. J2 (Accession No. AGC59908) and had a similar structural domain (an eight-stranded β/α barrel with a pronounced active-site cleft at the C-terminal end of the β-barrel) with the GH18 (glycosyl hydrolase, family 18) type II chitinase. However, *C. meiyuanensis* SYBC-H1 CHI2 protein was closest to the chitinase from *Chitinophaga pinensis* (Accession No.WP_012793147) and had the same catalytic domain as that of the glycoside hydrolase family 19 chitinase.

### 2.2. Structural Prediction of CHI Chitinase

**Figure 2:**
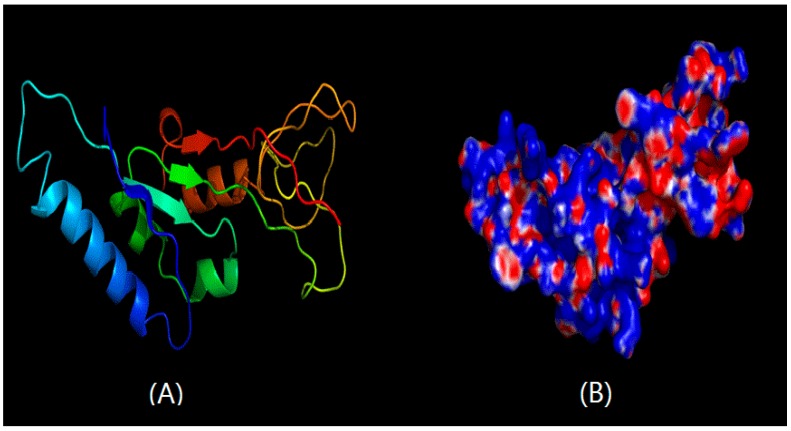
The predicted ribbon-model structure (**A**) and surface (**B**) of CHI1 chitinase from SYBC-H1.

**Figure 3:**
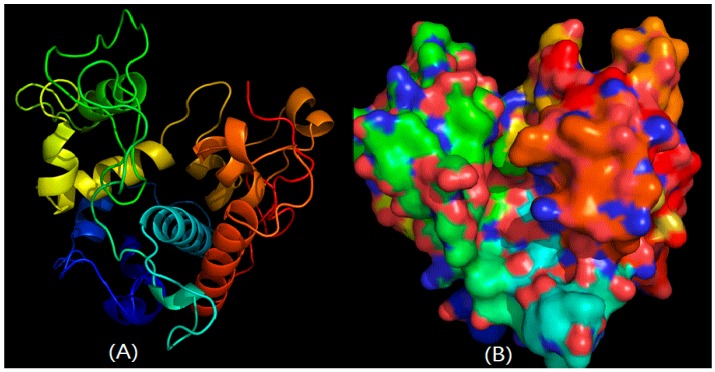
The predicted ribbon-model structure (**A**) (The PDB number is 2Z38) and surface (**B**) of CHI2 chitinase from SYBC-H1. Solvent-accessible surface representation colored by residue type (acidic residues red, basic residues blue, polar residues green, nonpolar residues white).

The reports were produced by SWISS-MODEL, using the structure of chitinase from *Vibrio harveyi* as a template for *C. meiyuanensis* SYBC-H1 CHI1 protein, and the structure of chitinase from *Carica papaya* as a template for the *C. meiyuanensis* SYBC-H1 CHI2 protein, since they share 51.03% sequence identity on the protein level. As shown in Figure 2A, CHI1 chitinase was mainly composed of five α and three β-sheets in the 3D structural prediction. The surface of CHI1 chitinase from SYBC-H1 is shown in Figure 2B. However, CHI2 chitinase consisted of 12 α without any sheets, indicating a stronger interaction and a more stable structure (Figure 3A). The surface of CHI2 chitinase is shown in Figure 3B. Additionally, the impact structure around the active center may suggest that both chitinases have a wide range of substrates. The result of surface showed that a higher proportion of base amine acid was in both CHI1 and CHI2 chitinases, indicating that the chitinases may have net positives in the neutral pH range and have the capability to efficiently adsorb negatively charged substrates.

### 2.3. Expression, Purification of CHI2 Protein in E. coli

**Figure 4:**
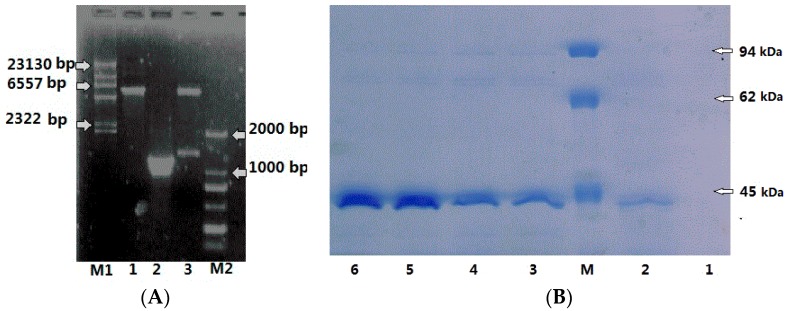
(**A**) Identification of plasmids constructed in this study (**M1**: λDNA/*Hind* III marker; **M2**: DL 2000 marker; **Lane 1**: pET28a vector; **Lane 2**: PCR amplicon; **Lane 3**: Recombinant plasmid); (**B**) SDS-PAGE analysis of induced *E. coli* BL21 cells carrying the pET28a-*CHI2*. (**M**: Protein marker, **Line 1**: Before induction; **Line 2**: Induction for 3 h; **Line 3**: Induction for 6 h; **Line 4**: Induction for 9 h; **Line 5**: Induction for 12 h; **Line 6**: Induction for 24 h).

Two experiments were carried out to express *CHI1* and *CHI2*, but *CHI1* was not successfully expressed. In order to attain the amount in expressed in CHI2 protein in *E. coli*, a pET28a expression vector was constructed with the PCR amplicon of *CHI2* gene (1149 bp) by *Xho* I and *Hind* III restriction sites. As shown in Figure 4A, the amplicon was successfully ligated into the expression vector in the 1% agarose.

The *E. coli* BL21 cells containing recombinant plasmid were cultured under the conditions of 30 °C and 200 rpm in 1 L Luria-Bertani medium containing 50 μg/mL kanamycin and induced with 1 mM IPTG (isopropyl β-d-1-thiogalactopyranosid) for 3 h of incubation (A_600 nm_ = 0.6). Protein expression in the induced *E. coli* BL21 was analyzed by SDS-PAGE analysis at intervals of 3 h. As shown in Figure 4B, CHI2 protein began to express after 6 h of induction, and the maximum accumulation of CHI2 protein occurred at 12 h. The chitinase activity of the supernatant was 0.38 U/mL after 6 h induction, indicating the expressed CHI2 chitinase was active on colloid chitin.

### 2.4. Characterization of Purified Chitinase

In this study, the chitinase was purified using Sephadex G-100 chromatography, resulting in 20.9% yield and 10.2-fold purification. The purification steps are summarized in Table 1.

### 2.5. Effects of Temperature and pH on the CHI2 Chitinase

**Figure 5:**
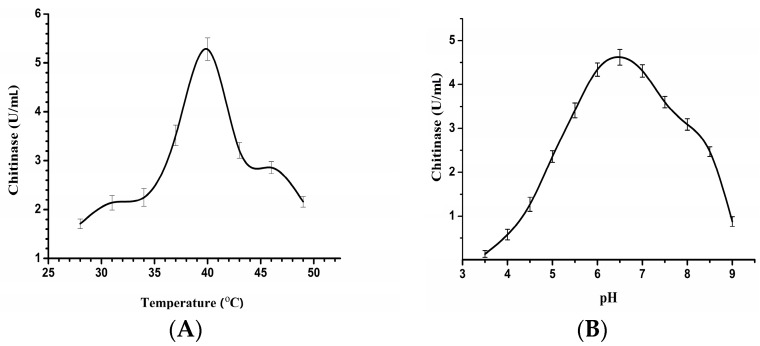
(**A**) Effect of temperature on the activity of chitinase from *C. meiyuanensis* SYBC-H1; (**B**) Effect of pH on the activity of chitinase from *C. meiyuanensis* SYBC-H1.

As shown in Figure 5A, the optimum temperature for the chitinase activity was 39 °C. Examination of their activities at temperatures ranging from 28 to 49 °C revealed it to be highly thermostable, as they retained nearly half of their activity (46.5%) at 30 °C for 120 h incubation. There residual enzyme activity was above 90% after 24 h of incubation at 49 °C, and had a half-life time of about 60 min at 60 °C, indicating its good thermal stability as compared to some other chitinases (Table 2). The CHI2 chitinase was active at the pH ranging from 3.5 to 9.0 with optimum pH of 6.5 (Figure 5B), similar to that for the chitinase from *C. tainanensis* [15]. A residual enzyme activity of 21% was obtained at pH 5.0 for 150 h of incubation. The high stability of CHI2 chitinase in response to temperature and pH makes it a good candidate for biotechnological applications involving the bioconversion of chitin waste into glucosamine products.

### 2.6. Effects of Metal Ions and Chemicals on the CHI2 Chitinase Activity

**Figure 6:**
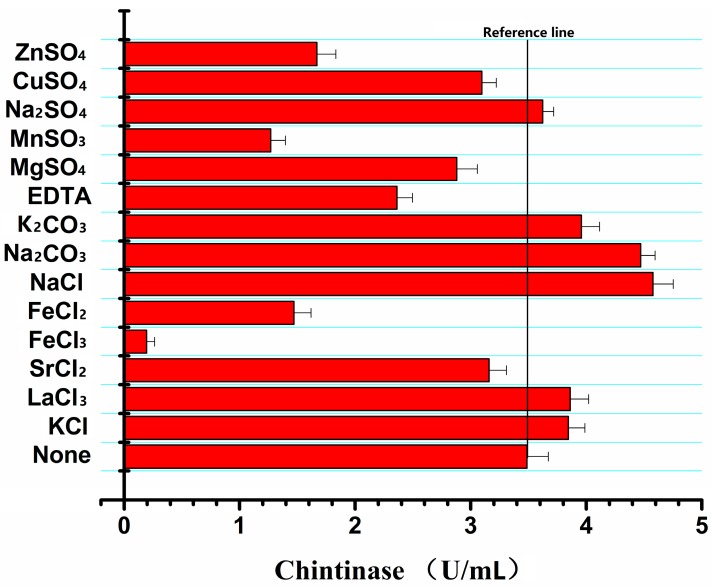
Effects of metal ion on the activity of chitinase from *C. meiyuanensis* SYBC-H1.

As shown in Figure 6, metal ions could markedly affect the activity of *CHI2* chitinase. The chitinase activity was completely inhibited by 10 mM Fe^3+^ and strongly inhibited by Fe^2+^ and Zn^2+^. However, Na^+^ and K^+^ significantly promoted chitinase activity by 31.4% and 14.3%. Effects of various chemicals on chitinase activity were also investigated. As shown in Table 3, V_c_, V_B6_, coenzyme, and polyethylene exhibited little relative effect on the stability of chitinase, whereas glutamine, cyclodextrin, galactose and soluble starch had a negative effect. Additionally, a notable phenomenon was that EGTA, EDTA and β-mercaptoethanol had significantly increased the stability of chitinase, particularly the β-mercaptoethanol. After 24 h of reaction, the enzyme activity of chitinase samples containing β-mercaptoethanol was higher than the control and remained 42% activity after 120 h while the control activity was almost lost. The improved enzyme stability could be attributed to protection of the protein free sulfhydryl from being oxidized by β-mercaptoethanol.

### 2.7. Kinetic Study and Chitin Hydrolysis

In order to investigate the ability of the chitinase to degrade chitin oligosaccharides and its affinity with the substrate, the kinetic constant of the purified chitinase was determined according to the method mentioned in Section 2.4, the *K*_m_ and *V*_mas_ values for the CHI2 chitinase were 23.0 mmol/L and 9.1 mM/min, respectively. Many factors may impact *K*_m_, in particular the substrate is an important factor. *p*-NP-GlcNAc was used in this study as the substrate, which may have been the reason for the higher *K*_m_ than literature values, in addition to other factors.

**Figure 7:**
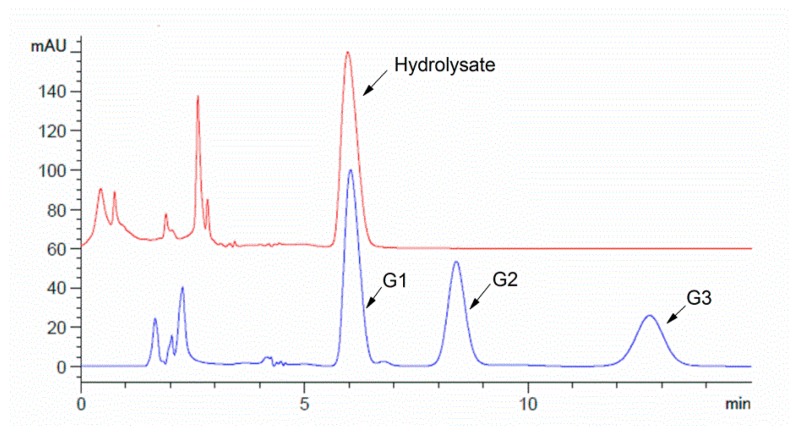
HPLC analysis of the hydrolyzed products of chitin (G1, G2 and G3 represent the standard sample of GlcNAc, Chitobiose and Chitotriose.

Possible products from chitin hydrolysis by the chitinase were analyzed using HPLC (High Performance Liquid Chromatography) after 12 h incubation and the results are shown in Figure 7. The monomer product GlcNAc was the only product detected, unlike the previous studies [5,17,18,19,20] in which dimer and trimer products were also observed, indicating incomplete conversion of chitin. The data obtained from HPLC analysis indicated that the CHI2 protein expressed by *E. coli* BL21 was an exo-hydrolytic *N*-acetyl glucosaminidase.

Chitin is usually thought as a renewable polysaccharide and an organic nitrogenous substance that is only preceded by cellulose and protein in abundance [21,22]. Although many microbial chitinases have been studied and characterized, the diversity of applications, molecular mechanisms, and optimal catalytic conditions are yet to be identified. Exploration of microbial diversity may help to find new enzymes with novel properties. Up to now, a number of attempts have been made to clone and express genes from many organisms (Table 4).

The protein structure determines its function. Protein 3D structure prediction involves predicting the 3-dimensional folding configurations from its amino acid sequence, and it helps to interpret the catalytic mechanism of chitinase. In this study, two *CHI* genes from *C. meiyuanensis* SYBC-H1 were cloned, respectively consisting of one 497 bp encoding a protein of 166 aa, and the other 1149 bp encoding a protein of 383 aa. The cloning of the *CHI2* chitinase, 3D structural prediction of CHI2 chitinase and expression in *E. coli* BL21 cells were investigated in this study for the first time. Overall, the findings indicate that CHI2 protein is endowed with a number of promising properties that are highly valuable for chitinase fundamental research and bioconversion of chitin waste. The deduced amino acid sequence of CHI1 showed high similarity (94%) with chitinase of *Staphylococcus* sp. J2 (JQ929768.1), and *CHI2* 97% with chitinase of *Chitinophaga pinensis* DSM 2588 (CP001699.1). The 3D structures of two chitinase cloned were simulated by homology modeling approach and the charge distribution of the surface was calculated. According to Figure 2A and Figure 3A, *CHI1* mainly contains four α-helices and three β-strands, while CHI2 contains 10 α-helices and there is only one β-stand. According to Figure 3, CHI2 chitinase mainly contains α-helices that intertwine with each other, therefore possibly having better stability. Both sides of the major groove of the active center are primarily irregular structures, thus the selectivity of CHI is not strong and may utlize substrates of diverse structures. It is evident that there are more negatives on the protein based on the simulated distribution of all charges, and there exists a concentrated area of partial positives, while the activity-related groove is dominated by negatives. In a neutral environment, the active center is expected to be a concentrated area of negatives. Therefore, a substance needs to be positive or neutral to be an active substrate of CHI2. It may also be speculated that it is better to be purified by cation exchange resin, as there is a partial concentration of positives.

Some chitinases were known to possess extremely stable protein folding that could be resumed after denaturation in the presence of SDS and reducing agent, whereas some chitinases could be renatured only if denatured in the presence of SDS alone [39]. Shown in Table 3, CHI2 incubated in the presence of β-mercaptoethanol exhibited greater activity after 24 h, which may suggest the renaturation of the deactivated enzyme. This result may indicate that the presence of disulfide bonds was essential for resuming the enzyme’s activity which is in agreement with the proposed formation of intramolecular disulfide linkage of chitinase 3D structure. There are 13 unpaired Cys residues in chitinase, and β-mercaptoethanol has a positive effect on the stability of chitinase. It is proposed that the β-SH group influences the redox status of the Cys residue in chitinase and further impacts its activity.

Kinetic parameters for CHI2 were determined using *p*-NP-GlcNAc concentrations ranging from 50 to 250 μM, and the *K*_m_ and *V*_max_ values for the CHI2 chitinase were 23.0 mM and 9.1 mM/min, respectively. A chitinase (*ChiA*) from *Bacillus licheniformis* was studied [5], reporting a *K*_m_ and *V*_max_ of 0.03 ± 0.003 mM and 0.28 ± 0.063 mM/min, respectively. In comparison, CHI2 chitinase may be an enzyme favorable for industrial uses with relatively high substrate concentrations.

## 3. Materials and Methods

### 3.1. Microorganism and Cultivation

Strain *C. meiyuanensis* SYBC-H1 (NCBI accession No.: GQ981314, CGMCC3438, ATCCBAA-2140) was a stock culture that had been previously isolated from the soil samples [12]. *E. coli* JM109, BL21, Vector pET28a, and Vector pMD18-T were purchased from TaKaRa Bio Co., Tokyo, Japan. Bacterial DNA extraction kits, plasmid recovery kits, DNA gel recovery kits, and primers were obtained from Saibaisheng Gene Co., Beijing, China. All other chemicals and solvents were of analytical grade and purchased from local suppliers.

A seed culture medium (g/L) containing 2.0 glucose, 4.0 peptone, 0.7 KH_2_PO_4_, 0.5 MgSO_4_·7H_2_O, 0.3 K_2_HPO_4_, and 0.02 FeSO_4_·7H_2_O was used to inoculate *C. meiyuanensis* SYBC-H1 and cultivate at a temperature of 37 °C. An *E. coli* strain was cultured in LB medium supplemented with ampicillin (100 μg/mL) at 30 °C for 24 h. Isopropyl β-d-1-thiogalactopyranoside (IPTG) (1 mmol/L) was added to the medium to detect the recombinant plasmids.

### 3.2. Cloning of CHI1 and CHI2 Gene

*C. meiyuanensis* SYBC-H1 was grown in seed culture medium for 8 h at 37 °C under shaking at 200 rpm, and then the cells were harvested by centrifugation at 4000× *g* for 10 min and used for genomic DNA isolation using the bacterial DNA extraction kit (Saibaisheng Gene Co.). The isolated DNA was confirmed using 1% agarose gel electrophoresis. Subsequently, the chitinase encoding gene *CHI* was amplified from genomic DNA of *C. meiyuanensis* SYBC-H1. The primers (5′-GGATCCATGAGTATAAACGCAGCAGG-3′, 5′-CTCGAGTTATTTTATGCGGATGATC-3′ and 5′-GGATCCGTCGACATCGACTGGGAG-3′, 5′-CTCGAGGCCGGTCCAGCCGCTACCGTAGAAG-3′) were designed according to a similar gene sequence obtained from NCBI (http://blast.ncbi.nlm.nih.gov/Blast.cgi) using the DANMAN 6.0 software. The components of PCR reaction (50 μL) included the following: primers (10 μmol/L), each at 1 μL; 4 μL dNTPs (2.5 mmol/L); 2 μL template DNA; 0.5 μL Taq DNA Polymerase; 5 μL 10× Taq PCR buffer, and 36.5 μL deionized (DI) water. PCR reaction was performed on a 96 well thermal cycler with a running program of 95 °C for 5 min (first cycle), 94 °C for 50 s, 53 °C for 1 min, 72 °C for 1 min (34 cycles) and the final extension for 5 min at 72 °C. The purified PCR amplicon was ligated into an expression vector, pMD18-T, and then transformed into *E. coli* JM109 cells. The *CHI* gene was isolated using a Gene JET plasmid Miniprep kit (Beijing Dingguo Biological Technology Co., LTD., Beijing, China), and then sequenced by BGISEQ Co., Beijing, China. Finally, the amplified fragment was again ligated into an expression vector, pET28a, using *BamH* I and *Hind* III restriction sites. Then, it was transformed into the chemically competent *E. coli* BL21 cells.

### 3.3. Sequence Analysis and Structure Prediction

The *CHI* gene sequence was translated into a protein sequence, which was compiled and compared with those in the GenBank database by the BLAST program. Multiple sequence alignments for proteins were created using Clustalx software (version 2.1, University of Tokyo, Human Genome Center, Tokyo, Japan) via default values. The phylogram was conducted by MEGA software (version 5.05, Arizona State University, Tempe, AZ, USA), and the bootstrap values (%) obtained with 1000 bootstrap re-samplings were shown at branching points. The 3D model was generated by homology modeling methods on SWISS-MODEL workspace (http://swissmodel.expasy.org/). The accessible surface area was calculated by the Discovery Studio (version 2.5, Accelrys, San Diego, CA, USA).

### 3.4. Kinetic Methods

Chitinase characterization was performed with *p*-NP-GlcNAc as a substrate: 50 μL enzyme solution was added to 1.95 mL *p*-NP-GlcNAc (0.25 mmol/L) in 50 mM sodium phosphate buffer solution (SPBS, pH 7.0) at 37 °C for a 10 min incubation. The reaction was terminated by adding 2 mL NaOH (0.5 mol/L). The release of *p*-nitrophenol from *p*-NP-GlcNAc was measured by recording the absorbance at 410 nm and then converted via the *p*-nitrophenol standards (20–100 μmol/L). One unit of chitinase activity was defined as the amount of enzyme required to release 1 μmol *p*-nitrophenol from the substrate per minute at 37 °C. The chitinase activity was assayed in triplicate.

The protein concentration was determined by the Bradford method [40] with bovine serum albumins as the standard.

### 3.5. Purification of CHI Chitinase

The *E. coli* BL21 expression cells were grown in 1 L LB medium containing 50 μg/mL kanamycin and induced with 1 mM IPTG (A_600 nm_ = 0.6) at 30 °C and 200 rpm for 3 h incubation. The culture was centrifuged at 4000× *g* for 15 min, with the supernatant collected, and the cells were then precipitated with ammonium sulfate (90 g/g) and left to stand overnight. The precipitate was collected by centrifugation at 10,000× *g* for 20 min and dissolved in 5 mL 50 mmol/L SPBS (pH 6.8). The solution was dialyzed to remove ammonium sulfate in the same buffer overnight. The dialyzed sample was passed through a DEAE-cellulose column (2.5 × 30 cm) equilibrated with 20 mM SPBS (pH 6.8). The fractions containing chitinase activity were pooled and concentrated using ultrafiltration tubes (molecular weight cut off 8000). The collected solution was separated by a Sephadex G-100 (1.6 × 90 cm) at the flow rate of 0.5 mL/min (20 mmol/L SPBS, pH 6.5). The active fractions were collected, desalted by dialysis against DI water and then lyophilized. All of the above procedures were conducted at 4 °C, except column separation

### 3.6. Characterization of CHI Chitinase

The protein was loaded onto SDS-PAGE gels using 5% stacking gel and 12% resolving gel in Tris-glycine buffer (pH 8.3), according to the method of Laemmli [41]. The molecular mass of the enzyme was estimated by staining with Coomassie Brilliant Blue G-250 (Solarbio, Beijing, China).

The effects of pH on enzyme activity were determined by incubating chitinase at different pH levels (3.5–9.5) and assaying the enzyme with the aforementioned method. pH stability was determined after incubating the purified chitinase at different pH values ranging from 4.0 to 8.0 without substrate, and the residual activity of the enzyme was calculated in the form of percentage of residual chitinase activity at the optimum pH.

Effect of temperature on purified enzyme activity was studied by incubating the reaction mixtures at different temperatures ranging from 28 to 49 °C at 3 °C intervals. As to the thermostability, it was measured by pre-incubating the chitinase at temperatures ranging from 25 to 65 °C at 10 °C intervals for 1 h, and then the residual activity was assayed using *p*-NP-GlcNAc as a substrate and calculated in the form of percentage residual chitinase activity at the optimum temperature.

Effects of metal ions on enzyme activity were investigated by adding Fe^3+^ (FeCl_3_), Mg^2+^ (MgSO_4_), Mn^2+^ (MnSO_4_), Cu^2+^ (CuSO_4_), Zn^2+^ (ZnSO_4_), Fe^2+^ (FeCl_2_), K^+^ (KCl), and Na^+^ (NaCl), respectively, into the reaction systems at the final concentration of 10 mM. Chemical reagents, V_c_, V_B6_, coenzyme, soluble starch, glutamine, polyethylene, cyclodextrin, galactose, EGTA, EDTA, dithiothreitol, and β-mercaptoethanol were also investigated at 10 mM for their effects on enzyme stability. The chitinase activity was measured using the method described above. The Michaelis-Menten constant (*K*_m_) and maximum velocity (*V*_max_) of purified chitinase were calculated graphically using the Lineweaver-Burk method, with the substrate *p*-NP-GlcNAc concentrations ranging from 50 to 250 μM in 50 mM SPBS (pH 7.0) at 37 °C.

### 3.7. Hydrolytic Analysis

The reaction system containing 0.9 U purified chitinase and 100 mg chitin powder in 10 mL SPBS (25 mM, pH 7.0) was conducted at 37 °C and 100 rpm for 12 h of incubation. The enzymatic hydrolysate was analyzed by HPLC. Samples were separated on an Alltima HP HILIC Column (4.6 mm × 250 mm) at a flow rate of 1.0 mL/min. The chromatography was performed at 40 °C. The hydrolyzed products were detected by monitoring absorbance at 210 nm with the mobile phase composed of acetonitrile and water (80:20, *v*/*v*).

## 4. Conclusions

In this research, a report that two *CHI* genes from *Chitinolyticbacter meiyuanensis* SYBC-H1 encoding chitinases were identified and their protein 3D structures were predicted. *CHI2* chitinase was expressed in *E. coli* BL21 cells, and this protein was purified by ammonium sulfate precipitation, DEAE-cellulose, and Sephadex G-100 chromatography. Optimal activity of CHI2 chitinase occurred at a temperature of 40 °C and a pH of 6.5. The presence of metal ions Fe^3+^, Fe^2+^, and Zn^2+^ inhibited CHI2 chitinase activity, while Na^+^ and K^+^ promoted its activity. Furthermore, the presence of EGTA, EDTA, and β-mercaptoethanol significantly increased the stability of CHI2 chitinase. The CHI2 chitinase was active with the *p*-NP-GlcNAc. Overall, our results demonstrated CHI2 chitinase with its remarkable biochemical properties is suitable for bioconversion of chitin waste.

## Figures and Tables

**Table 1:** Purification steps of CHI2 chitinase.

Purification Steps	Total Activity (U)	Total Protein (mg)	Specific Activity (U/mg)	Yield (%)	Purification Fold
Crude enzyme	239.0	391.1	0.6	100	1
Ammoniumsulfate	161.1	106.0	1.5	67.4	2.5
DEAE-cellulose	90.3	24.5	3.9	37.8	6.0
Sephadex G-100	50.0	8.0	6.2	20.9	10.2

**Table 2:** Comparison between the properties of CHI2 chitinase and those of other typical chitinases.

Chitinase	Microorganism	Optimum pH	Optima Temperature (°C)	Half-Life Time (°C)	*M*_w_ (kDa)	Ref.
*CHI2*	*C.meiyuanensis* SYBC-H1	6.5	40	60 min (60 °C)	42	This study
*Chi*	*Bacillus* sp. NTCU2	7	60	30 min (60 °C)	36.5	[11]
*Chi*	*Bacillus licheniformis* MB-2	6	70	80 min (60 °C)	67	[8]
*Chi*	*Streptomyces* RC1071	8	40	60 min (60 °C)	70	[16]
*Ch501*	*Streptomyces* sp. CS501	7	60	60 min (55 °C)	43	[10]
*ChiA*	*Pseudoalteromonas* sp. DL-6	8	20	60 min (40 °C)	110	[2]

**Table 3:** Effects of various reagents on CHI2 chitinase with colloidal chitin as the substrate.

Chemicals	0 h	24 h	48 h	72 h	96 h	120 h
No addition	100	95.80	75.18	54.43	43.76	15.09
V_c_	100	96.05	71.92	42.50	22.82	16.17
V_B6_	100	91.18	64.73	58.45	41.47	15.43
Coenzyme	100	95.13	59.81	57.22	39.14	20.03
Glutamine	100	88.37	49.28	32.72	29.29	16.62
Polyethylene	100	76.62	60.45	56.36	41.36	36.91
Cyclodextrin	100	76.93	50.26	33.79	20.16	16.97
Galactose	100	96.59	36.06	16.30	13.12	12.34
Soluble starch	100	75.15	86.01	24.23	15.71	12.62
EGTA	100	94.87	84.60	62.91	63.06	44.50
EDTA	100	76.73	58.19	54.42	42.77	44.56
Dithiothreitol	100	94.46	66.25	52.08	32.13	18.22
β-Mercaptoethanol	100	127.50	101.33	86.54	73.83	46.47

**Table 4:** Selected organisms with chitinase gene cloned.

Gene Sources	Gene Name	Year	Reference
*Vibrio vulnificus*	*pATW501*, *502*, *503*	1986	[23]
Rice	*Rice Ch t*	1991	[24]
*Altermonas* sp. strain O-7	*pCHI997*	1993	[25]
*Trichoderma harzianum*	*CHIT42*	1994	[26]
Human	*Chitotriosidase*	1995	[27]
*Coccidioides immitis* CF	*CF*	1996	[28]
*Aspergillus nidulans*	*ChiA*	1998	[29]
*Streptomyces thermoviolaceus* OPC520	*Chi25*	2000	[30]
*Heterodera glycines*	*designated Hg-chi-1*	2002	[31]
*Paralichthys olivaceus*	*fChi1*, *fChi2* and *fChi3*	2004	[32]
*Trichoderma atroviride* strain P1	*ech30*	2006	[33]
*Pichia pastoris*	*chi58*	2009	[34]
*Limonium bicolor*	*Lbchi31*	2010	[35]
*Aphanomyces astaci*	*CHI1*, *CHI2* and *CHI3*	2012	[36]
*Ostrinia*	*OfCht5*	2013	[37]
*Bacillus licheniformis* LHH100	*ChiA-65*	2015	[38]
